# Poverty Vulnerability and Health Risk Action Path of Families of Rural Elderly With Chronic Diseases: Empirical Analysis of 1,852 Families in Central and Western China

**DOI:** 10.3389/fpubh.2022.776901

**Published:** 2022-02-14

**Authors:** Ying Ma, Qin Xiang, Chaoyang Yan, Hui Liao, Jing Wang

**Affiliations:** ^1^Department of Health Management, School of Medicine and Health Management, Tongji Medical College, Huazhong University of Science and Technology, Wuhan, China; ^2^The Key Research Institute of Humanities and Social Science of Hubei Province, Huazhong University of Science and Technology, Wuhan, China; ^3^Institute for Poverty Reduction and Development, Huazhong University of Science and Technology, Wuhan, China

**Keywords:** poverty vulnerability, health risk, rural elderly, health poverty, chronic disease

## Abstract

Health poverty has become the most important cause of poverty and return to poverty. Understanding the health risk factors and action paths of poverty in families of rural elderly with chronic diseases is important to alleviate return to poverty because of illness. This study selected families with at least one elderly member (over 60 years old) with chronic diseases (sample size was 1,852 families) in two provinces and four counties in central and western China. The three-stage feasible generalized least square method was adopted, and the appropriate poverty line standard was selected to measure the poverty vulnerability index. A poverty vulnerability index ≥50% was considered to indicate vulnerability. The poverty vulnerability index and actual income status were combined to classify the samples. A structural equation model was established to explore the path of each health risk factor on the entire sample and various types of poverty vulnerabilities. The mean poverty vulnerability of 1,852 families was 0.5974 ± 0.25213, and among which, 1,170 households had a poverty vulnerability value ≥0.5, accounting for 63.17% of the entire sample. The incidence of poverty was higher among people with low vulnerability to poverty. Health shock was the direct cause of poverty for people with potential and avoidance poverty. The mediating roles of family and community significantly differed in various types of poverty vulnerability. The social and economic environment in rural areas should be enhanced in a diversified manner, and the income-generating ability of rural households should be improved based on actual local conditions. Moreover, the prevention and control of poverty vulnerabilities should be diversified and targeted. Policies implemented should be based on people and localities, the causes of poverty and returning to poverty, and the types of poverty vulnerabilities. The use efficiency of medical insurance should be further improved, and the responsibility of medical insurance targeted poverty alleviation must be clarified.

## Introduction

According to the World Health Organization (WHO), health is a “state of complete physical, mental, and social wellbeing and not merely the absence of disease or infirmity” (1). Health shocks constitute a sudden deterioration in the state of an individual's health, caused by an illness and/or injury, which can negatively affect personal and family welfare and cause health poverty (2–4). Health poverty is a type of medical expenditure poverty caused by too low affordability and too high demand for medical services (4). Low- and middle-income countries including developed countries are affected by poverty caused by illness (5). With the rapid development of China's economy and society and the strong support of targeted Poverty Alleviation Policies in recent years, China's fight against poverty has won an all-round victory by the end of 2020. All 98.99 million rural poor people under the current standard have been lifted out of poverty, and the arduous task of eliminating absolute poverty has been completed (6). Although absolute poverty has been solved, relative poverty still exists. According to the data of the Poverty Alleviation Office of the State Council in 2017, 42.3% of the poverty-stricken households in China suffered poverty because of illness or returned to poverty (7).

Families of the elderly with chronic diseases in rural areas are the key population with a high risk of health poverty. The unique physical characteristics, psychological characteristics, disease patterns, pension patterns, accessibility of medical resources, and socio-economic status of the elderly with chronic diseases in rural areas determine that they are vulnerable to health hazards and poverty. The rural elderly are suffering from poverty because of high-intensity labor participation, malnutrition, minor diseases dragged into serious diseases, and others. In addition, the high demand for medical services and low ability to pay lead to poverty because of diseases, causing the elderly to fall into a vicious cycle of poverty and diseases (8). Compared with the urban elderly, the rural elderly have a lower quality of life (9) and poorer accessibility to health services (10). Moreover, chronic diseases bring a heavier disease economic burden to the rural elderly than the urban elderly (11, 12), explaining 7.9% of the causes of poverty (13). With the rapid development of aging and an empty nest, the health poverty of the rural elderly cannot be ignored.

Health poverty has brought a lasting and profound negative impact on the rural elderly and their families. On the one hand, the rural elderly with health poverty have low health capital stock and insufficient health ability. Hence, they are faced with a great probability of health risk impact, which directly leads to a substantial increase in medical expenditure (14, 15). In addition, a negative correlation exists between health level and health income, and the higher the income inequality, the lower the health level (16). Furthermore, poverty because of illness exerts a significant dynamic impact on long-term poverty in the future. The probability of poverty because of illness is at least 2.2 times higher than that of non-poor households in the next 2 years (17). On the other hand, previous studies have showed that a low socio-economic level inhibits the enthusiasm of residents to invest in their own health. Compared with non-poor families, poor families are more vulnerable to the impact of diseases, and their ability to disperse economic risks caused by diseases is weaker (18, 19). Moreover, the large increase in medical expenditure of the elderly inevitably affects the investment in education and health of future generations. This scenario hinders the improvement of future generations in terms of learning, working ability, and health and causes the intergenerational transmission of poverty (20, 21). The health vulnerability of the elderly is closely related to the economic vulnerability of the family, forming a vicious cycle of “poverty caused by illness and disease caused by poverty.” Health poverty results in economic and health losses. Therefore, the health risk factors and action path of poverty in families of rural elderly with chronic diseases should be understood, and intervention measures must be implemented to avoid poverty consequences.

Health poverty vulnerability refers to the possibility of an individual or family falling into a low level of welfare after being impacted by health-related risks (22, 23). Health poverty vulnerability is the risk of falling into poverty in the future (24, 25) and also refers to the risk that the current state of poverty will continue into the future (26, 27). Vulnerability to poverty is seen widely as a better future-oriented/forward-looking welfare measure (28–31). Examining health poverty from the perspective of poverty vulnerability is helpful to understand health poverty dynamically and establish forward-looking Poverty Alleviation Policies. Most related studies in recent years only analyzed the current situation and influencing factors of health poverty vulnerability. Some studies analyzed the link between current health status and vulnerability to poor health (3, 32). These studies focused on after the occurrence of health risks and did not explore the path of action. Jiandong Chen measured rural poverty and vulnerability from five aspects: natural resources, human resources, physical assets, financial assets, and social resources (33). In addition, some studies on vulnerability mainly focused on the relationship between ecological vulnerability and poverty (34–37) or the relationship between food vulnerability and poverty (38, 39). At present, studies on the measurement of health poverty vulnerability are limited. Moreover, relatively few studies exist on the path of the health risk factors for poverty vulnerability.

Various health risk factors that affect the level of family welfare are analyzed to predict the probability of a family falling into poverty at a specific time in the future (40, 41). Clarifying the mechanism of health risk factors of poverty vulnerability is helpful for in formulating more accurate and efficient health poverty alleviation measures, to improve the efficiency and effect of poverty alleviation. Poverty alleviation should focus not only on the current situation but also on the risk groups (e.g., the highly vulnerable people suffering from serious diseases and chronic diseases) in the future. In addition, the most appropriate assistance measures should be chosen according to the action path of the risk factors. The objectives of this study are the following: (1) calculate the future poverty vulnerability based on the expected poverty vulnerability theory and explore the coherence between poverty vulnerability and poverty; (2) analyze the path of each health risk factor on the entire sample and various types of poverty vulnerabilities. China has made great achievements in health poverty alleviation and can be used as a model by other relatively underdeveloped countries and regions. This study provides targeted anti-poverty suggestions for long-term anti-poverty mechanisms in the future.

## Methods

### Data Sources

Data were obtained from an on-site survey conducted by the National Natural Science Foundation of China on rural elderly and their family members. In 2018, we surveyed rural elderly with chronic diseases and their family members over 60 years old in two provinces and four counties in central and western China. The survey used multi-stage stratified random sampling. Two national-level poverty-stricken counties were selected from each province. Five townships were randomly selected from each poor county, and three natural villages were randomly selected from each township. Each natural village randomly selected approximately 30 chronically ill elderly households for the household survey.

The survey content included personal and family characteristics, income and expenditure, health status and disease characteristics, lifestyle, accessibility and service experience of medical services, forms of sharing medical expenses, social networks, and social capital. The survey also focused on collecting other data related to health poverty risk indicators and poverty vulnerability measurement. After screening out at least one chronically ill elderly family over 60 years old and deleting samples with missing key variables, the final sample size for analysis was 1,852 families.

### Variables and Definitions

The variables for calculating poverty vulnerability were mainly divided into two levels: individual and family. At the individual level, five variables were selected: gender, age, marital status, education level, and self-rated health status of the main interviewee. At the family level, five variables were selected: number of family members, number of non-working-age family members, whether or not the family members are engaged in non-agricultural work, level of social security, and number of family members with chronic disease.

Health risk factors included three dimensions: health shock, family coping ability, and community health support system. Health shock included two dimensions: health physiological status and health economic burden. Family coping ability included three dimensions: family coping behavior, affordability of family assets, and family health protection status. The community health support system included two dimensions: accessibility of health services and availability of health services. The detailed coding of each variable is shown in Table 1.

**Table 1:** Health risk indicators and codes used.

**Variables**			**Health risk variables**	**Code**
Health shock	Health physiological status	Breadth of family health impact	Number of family members suffering from multiple chronic diseases	X1
			Number of disabled family members	X2
		Depth of family health impact	The nature of chronic disease of family members	X3
			Family member disability level	X4
	Health economic burden	Direct health burden	Health (including medical and nursing care) expenditure as a percentage of total household expenditure	X5
		Indirect health burden	Loss of income due to health	X6
Family coping ability	Family coping behavior	Coping initiative	Should be hospitalized but not hospitalized	X7
		Coping intensity	Number of hospitalizations per year	X8
			Annual number of hospitalization days per capita for patients with chronic diseases in the family	X9
	Affordability of family assets	Stock assets	Relative poverty line level of family income per capita	X10
		Potential assets	Percentage of effective family labor	X11
	Family health protection status	Basic medical insurance	Family basic medical insurance type	X12
Community health support system	Accessibility of health services	Space accessible	Distance to nearest medical service institution	X13
		Accessible services	Disability care methods	X14
	Availability of health services	Capability support	Chronic disease diagnosis and treatment institution level	X15

### Statistical Method

Based on the expected poverty vulnerability theory, the poverty vulnerability index of families of rural elderly with chronic diseases was calculated. Descriptive statistics were used to analyze the overall situation of the sample, and the rural elderly households were divided into four types according to the two dimensions of whether or not they are poor and whether or not they are vulnerable to poverty. Finally, the structural equation model was used to identify the path of health risk factors on poverty vulnerability.

### Calculation of Poverty Vulnerability

Given the use of cross-sectional data, this study was based on the expected poverty vulnerability theory. Chaudhuri's three-stage feasible generalized least squares (FGLS) method was used to estimate the logarithmic expectation of per capita income and the logarithmic variance of per capita income of rural elderly households with chronic diseases, thereby measuring its poverty vulnerability index (24). Specific steps are as follows:

The income density function was used to express the poverty vulnerability of family *i* at time *t*, which is the probability of a family to fall below the poverty line at time *t*+1, which can be expressed as Equation (1):


(1) $$\begin{array}{l} V_{i,t}=\int_{-\infty }^{z}f_{t}\left(Y_{i,t+1}\right)d\left(Y_{i,t+1}\right) \end{array}$$

where *f* is the density function, *i* and *t* are the individual and time identifiers, respectively, and *Y* is the level of welfare (replaced by income).

Second, the income function was estimated. Assuming that the income level of rural elderly with chronic illness at period *t*+1 is a function of personal characteristics at period *t*, the logarithm of the income at period *t*+1 is regressed to estimate, which can be expressed as Equation (2):


(2) $$\begin{array}{l} Ln\;Y_{ln+1}=X_{ln}\beta_{i}+e_{i} \end{array}$$

where *Y*_ln +1_represents the income level of family *i* of rural elderly with chronic illness at period *t*+1, and *X*_ln_ is a series of poverty vulnerability characteristic variables that affect future income. Combined with the literature and field survey interviews, the dependent variable of the income function was selected as the logarithm of per capita annual income of the sample household. The choice of independent variables was mainly divided into two levels: individual and family. At the individual level, five variables were selected: gender, age, marital status, education level, and ability of daily living (ADL) of the main respondent. At the family level, five variables were selected: number of the family population, number of people under the age of 16, number of people working in the family, whether or not family members participate in work other than agriculture, and whether or not any family members have received high school education and above. Regarding the residual square as the approximate value of the income variance $\sigma_{e,i}^{2}$, the residual square is used as the dependent variable to construct the regression model of the residual square $\sigma_{e,i}^{2}$ on the characteristic variables of the rural elderly with chronic diseases, expressed as Formula (3):


(3) $$\begin{array}{l} \hat{e_{i}^{2}}=X_{i}\theta +\gamma_{i} \end{array}$$

The weighted regression of the income logarithm, that is, the residual square, was performed to obtain the estimated quantities $\hat{\beta }$ and $\hat{\theta }$ of FGLS. Then, the expectation and variance of the future income logarithm were estimated, as shown in Equations (4) and (5):


(4) $$\begin{array}{l} Ê\left(\text{Ln}Y_{\ln}|X_{i}\right)=X_{i}\hat{\beta } \end{array}$$

(5) $$\begin{array}{l} \hat{V}\left({\text{Ln Y}}_{\ln}|X_{i}\right)=X_{i}\hat{\theta } \end{array}$$

The Pareto distribution is suitable to describe the income level of high-income groups, whereas the lognormal distribution is suitable to describe the income level of low-income groups.

Finally, the probability of family i falling into poverty at time t can be expressed as Equation (6):


(6) $$\begin{array}{l} \hat{V_{i,t}}=P\left(\text{Ln}Y_{i}<\text{Ln}Z|X_{i}\right)=\Phi \left(\frac{\text{Ln}Z-X_{i}\hat{\beta }}{\sqrt{X_{i}\hat{\theta }}}\right) \end{array}$$

where $\hat{V_{i,t}}$ represents the expected poverty vulnerability index, Φ(∙) represents the cumulative density function of the standard normal distribution, Z represents the poverty line, $X_{i}\hat{\beta }$ represents the expected value of the logarithm of per capita income, and $X_{i}\hat{\theta }$ represents the expected variance of the logarithm of per capita income.

Suppose the probability of falling into poverty in the future was higher than 50% as the criterion of vulnerability, that is, the probability of a family falling into poverty in the future was more than 50%. In this case, the family was considered poor and vulnerable.

### Poverty Vulnerability Calculation Standard

This study selected ¥3535 /person/year as the poverty line standard and calculated the poverty vulnerability of rural elderly families as a key variable for subsequent research. That is, the study combines the actual situation of the survey area and the survey sample and the researcher's professional background knowledge.

### Poverty Vulnerability Classification

**Figure 1:**
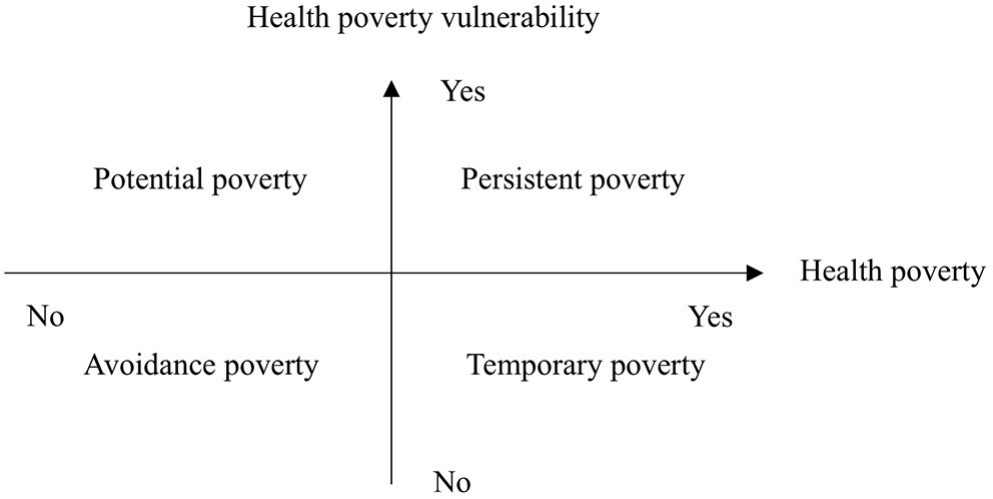
Dichotomies of health poverty and vulnerability.

The study combined the two dimensions of whether or not rural elderly households are poor and whether or not they are vulnerable to poverty, and divides the population into four categories, namely, persistent poverty (current poverty and poverty vulnerability), temporary poverty (current poverty but no poverty vulnerability), potential poverty (current poverty but poverty vulnerability), and avoidance poverty (current poverty and no poverty vulnerability) in Figure 1.

### Ethical Approval

The Ethics Committee of Tongji Medical College, Huazhong University of Science and Technology approved the research proposal (IRB: IORG0003571). All respondents provided informed consent before they were interviewed.

## Results

Results showed that the average poverty vulnerability of 1,852 households was 0.5974 ± 0.25213, and of which, 682 households had a poverty vulnerability value of <0.5, accounting for 36.83% of the entire sample. Moreover, 1,170 households had a poverty vulnerability value ≥0.5, accounting for 63.17% of the entire sample. In the coming year, more than 60% of rural elderly households may be at risk of poverty.

In this study, the household poverty vulnerability values were divided into 0–0.25, 0.25–0.5, 0.5–0.75, and 0.75–1.0 quartiles, which were, respectively, defined as low vulnerability, lower vulnerability, higher vulnerability, and high vulnerability.

As shown in Table 2, a significant difference existed between the quartile of poverty vulnerability and the incidence of poverty. People with low vulnerabilities had a high incidence of poverty.

**Table 2:** People with low vulnerabilities had a high incidence of poverty.

**Vulnerability quartile**	**Poverty**	**Non-poverty**	****χ** ^2^**
	**(n/%)**	**N(n/%)**	
0–0.25	68 (36.2)	120 (63.8)	9.288^*^
0.25–0.5	233 (47.2)	261 (52.8)	
0.5–0.75	225 (43.9)	288 (56.1)	
0.75–1.0	264 (40.2)	393 (59.8)	

* *P < 0.05*.

As shown in Table 3, the numbers of persistent, temporary, potential, and avoidance poverty households in rural elderly families were 489, 301, 681, and 381, respectively, accounting for 26.40, 16.25, 36.77, and 20.57% of the total sample. Potentially poor households accounted for the largest proportion, followed by persistently poor households. Only approximately one-fifth of them can really avoid poverty. This finding shows that the rural elderly in our country are vulnerable to poverty. Even the households that have not fallen into poverty in accordance with the current poverty line standards are likely to fall into poverty once they suffer a risk shock.

**Table 3:** Health risk characteristics of different types of poverty vulnerabilities.

**Variable**	**Persistent poverty**	**Temporary poverty**	**Potential poverty**	**Avoid poverty**	****χ** ^2^**
	**(n/%)**	**(n/%)**	**(n/%)**	**(n/%)**	
Total	489 (26.40)	301 (16.25)	681 (36.77)	381 (20.57)	
Number of family members suffering from multiple chronic diseases					14.889^*^
≤ 1	401 (82.00)	233 (77.41)	574 (84.29)	303 (79.53)	
2	82 (16.77)	67 (22.26)	95 (13.95)	74 (19.42)	
≥3	6 (1.23)	1 (0.33)	12 (1.76)	4 (1.05)	
Number of disabled family members					12.736^*^
≤ 1	446 (91.21)	271 (90.03)	644 (94.57)	359 (94.23)	
2	39 (7.98)	29 (9.63)	35 (5.14)	22 (5.77)	
≥3	4 (0.82)	1 (0.33)	2 (0.29)	0 (0.00)	
The nature of chronic disease of family members					19.678^*^
Common chronic diseases	368 (75.26)	209 (69.44)	555 (81.50)	281 (73.75)	
Major chronic diseases	121(24.74)	92 (30.56)	126 (18.50)	100 (26.25)	
Family member disability level					33.738^*^
No	287 (58.69)	177(58.80)	477 (70.04)	270 (70.87)	
Mild	160 (32.72)	106 (35.22)	156 (22.91)	93 (24.41)	
Moderate	17 (3.48)	5 (1.66)	22 (3.23)	7 (1.84)	
Severe	25 (5.11)	13 (4.32)	26 (3.82)	11 (2.89)	
Health (including medical and nursing care) expenditure as a percentage of total household expenditure					15.892
≤ 15%	131 (26.79)	57 (18.94)	194 (28.49)	94 (24.67)	
15–40%	127 (25.97)	78 (25.91)	192 (28.19)	107 (28.08)	
>40%	231(47.24)	166 (55.15)	295 (43.32)	180 (47.24)	
Loss of income due to health					8.225^*^
Yes	76 (15.54)	31 (10.30)	85 (12.48)	37 (9.71)	
No	413 (84.46)	270 (89.70)	596 (87.52)	344 (90.29)	
Should be hospitalized but not hospitalized					1.881
Yes	24 (4.91)	11 (3.65)	29 (4.26)	12 (3.15)	
No	465 (95.09)	290 (96.35)	652 (95.74)	369 (96.85)	
Number of hospitalizations per year					17.777^*^
≤ 1	329 (67.28)	212 (70.43)	456 (66.96)	299 (78.48)	
≥2	160 (32.72)	89 (29.57)	225 (33.04)	82 (21.52)	
Annual number of hospitalization days per capita for patients with chronic diseases in the family					41.690^*^
≤ 5 days	351 (71.78)	174 (57.81)	523 (76.80)	255 (66.93)	
5–10 days	37 (7.57)	26 (8.64)	44 (6.46)	31 (8.14)	
>10 days	101 (20.65)	101 (33.55)	114 (16.74)	95 (24.93)	
Relative poverty line level of family income per capita					152.855^*^
≤ 1	334 (68.30)	150 (49.83)	449 (65.93)	140 (36.75)	
1–1.5	56 (11.45)	37 (12.29)	74 (10.87)	45 (11.81)	
1.5–2	32 (6.54)	32 (10.63)	46 (6.75)	34 (8.92)	
>2	67 (13.70)	82 (27.24)	112 (16.45)	162 (42.52)	
Percentage of effective family labor					36.774^*^
≤ 1/3	297 (60.74)	157 (52.16)	471 (69.16)	250 (65.62)	
1/3–2/3	175 (35.79)	123 (40.86)	191 (28.05)	108 (28.35)	
>2/3	17 (3.48)	21 (6.98)	19 (2.79)	23 (6.04)	
Family basic medical insurance type					67.000^*^
Medical insurance for urban and rural residents	484 (98.98)	296 (98.34)	669 (98.24)	344 (90.29)	
Urban employee medical insurance	1 (0.20)	2 (0.66)	7 (1.03)	25 (6.56)	
Other	4 (0.82)	3 (1.00)	5 (0.73)	12 (3.15)	
Distance to nearest medical service institution					42.461^*^
≤ 10 min	132 (26.99)	108 (35.88)	269 (39.50)	168 (44.09)	
10–20 min	117 (23.93)	77 (25.58)	177 (25.99)	89 (23.36)	
20–30 min	85 (17.38)	40 (13.29)	90 (13.22)	40 (10.50)	
>30 min	155 (31.70)	76 (25.25)	145 (21.29)	84 (22.05)	
Disability care methods					95.082^*^
No disability	362 (74.03)	170 (56.48)	562 (82.53)	237 (62.20)	
Home care	118 (24.13)	116 (38.54)	108 (15.86)	125 (32.81)	
Institutional care and others	9 (1.84)	15 (4.98)	11 (1.62)	19 (4.99)	
Chronic disease diagnosis and treatment institution level					40.852^*^
Grassroots	313 (64.01)	230 (76.41)	408 (59.91)	268 (70.34)	
County level	79 (16.16)	44 (14.62)	154 (22.61)	70 (18.37)	
Above county level and others	97 (19.84)	27 (8.97)	119 (17.47)	43 (11.29)	

* *P < 0.05*.

Table 3 shows that except for the variables that should be hospitalized but not hospitalized, no statistical difference existed among the four types of poor and vulnerable households, whereas the other variables showed significant differences. Health risk indicators played different roles in various types of poverty vulnerabilities, and their risk expressions were different.

**Figure 2:**
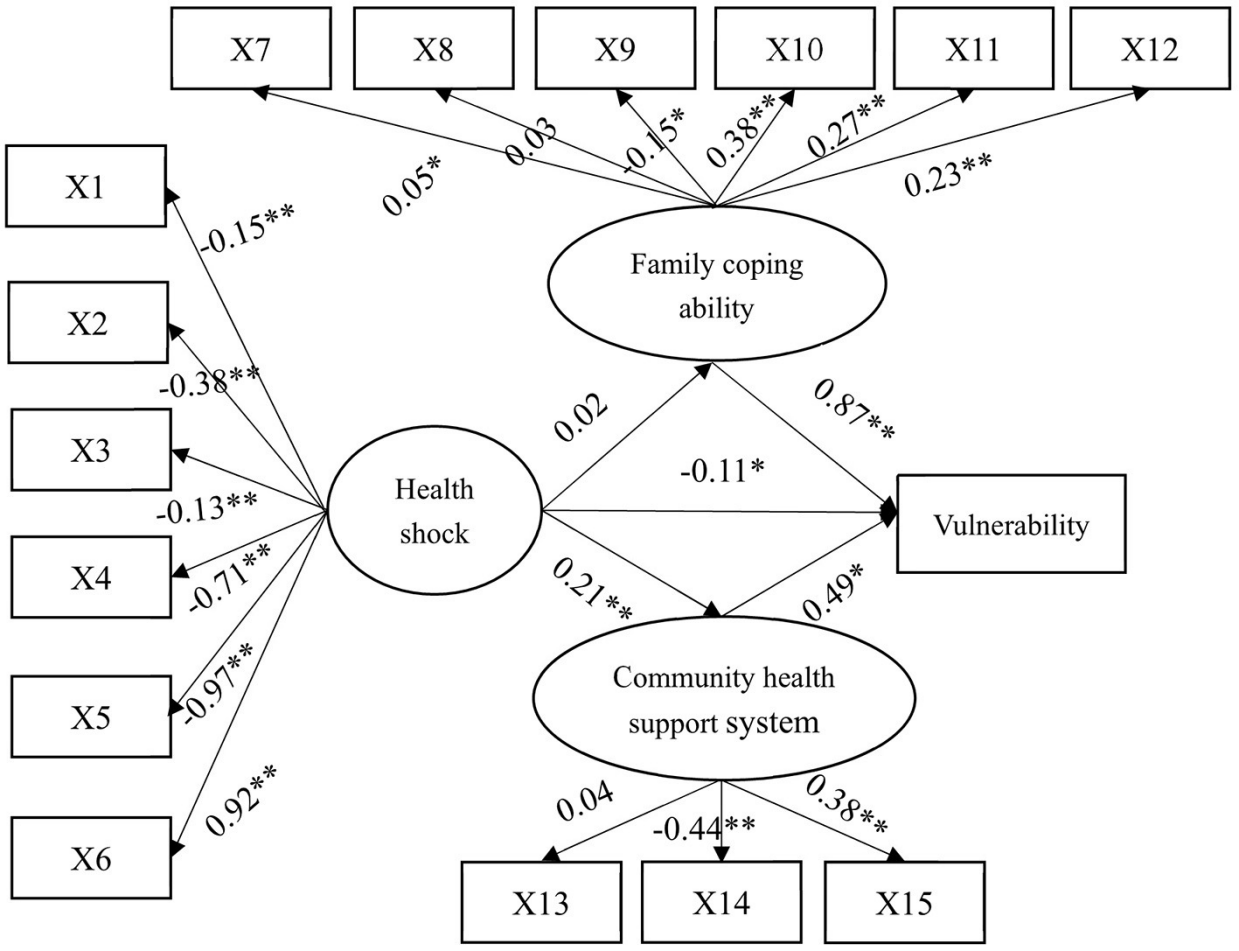
Full-sample structural equation model path diagram and standardized solution. *p < 0.05 **p < 0.01.

Figure 2 shows that the model fitting results of the entire sample show that the model fitting effect improved. Root mean square residual (RMR) = 0.03 <0.05, goodness-of-fit index (GFI) = 0.97>0.90, adjusted goodness-of-fit index (AGFI) = 0.96>0.90, parsimony goodness-of-fit index (PGFI) = 0.71>0.50, parsimony normed fit index (PNFI) = 0.57>0.50, and other commonly used fitting indexes indicate that the model fit well and can be used to analyze the path of health risk factors on elderly chronically ill families.

Table 4 shows the structural equation model analysis results of the entire sample and four grouped samples. The results of structural equation analysis of the whole sample show that the direct and indirect effects of health shock on the poverty vulnerability of rural chronic disease families are significant. The action direction of the two is opposite, and the final total effect is positive reinforcement. In addition, family coping ability and community health support system have a significant direct impact on the poverty vulnerability of rural chronic disease families, and both have a positive impact. For the whole sample, health impact can affect poverty vulnerability in two ways: one is the direct effect caused by various acute and chronic diseases, disability, mental disorders, and other diseases. Second, through the intermediary role of a family coping ability dimension and community health support system dimension, the health risk is transformed into economic risk and finally manifested as poverty vulnerability.

**Table 4:** Standardized effect statistics table.

**Sample**	**Path**	**Direct effect**	**Indirect effect**	**Total effect**
Full	Health shock- Vulnerability	−0.106^*^	0.125^**^	0.019^*^
	Family coping ability- Vulnerability	0.874^**^	——	0.874^**^
	Community health support system - Vulnerability	0.491^*^	——	0.491^*^
Avoidance poverty	Health shock- Vulnerability	0.338^**^	−0.144^**^	0.194^**^
	Family coping ability- Vulnerability	0.401^*^	——	0.401^*^
	Community health support system - Vulnerability	0.106^**^	——	0.106^**^
Potential poverty	Health shock- Vulnerability	0.131^*^	−0.225^**^	−0.095^**^
	Family coping ability- Vulnerability	−0.566^**^	——	0.566^**^
	Community health support system - Vulnerability	0.349	——	0.349
Temporary poverty	Health shock- Vulnerability	0.082	0.222	0.139^**^
	Family coping ability- Vulnerability	0.422	——	0.422
	Community health support system - Vulnerability	0.026	——	0.026
Persistent poverty	Health shock- Vulnerability	0.066	0.033	0.099^*^
	Family coping ability- Vulnerability	0.361^*^	——	0.361^*^
	Community health support system - Vulnerability	0.200^*^	——	0.200^*^

* *P < 0.05*,

** *P < 0.01*.

In addition, the family coping ability dimension has the greatest effect on the poverty vulnerability of rural chronic disease families. That is, the weaker the family coping ability, the more likely it is to have poverty vulnerability. Second, the role of the dimension of the community health support system is also more important. The worse the accessibility and availability of medical services, the more prone to poverty vulnerability. Among them, the role of disability care is the most prominent, indicating that service accessibility is the most important for the community support system.

The results of the structural equation model analysis of the poverty avoidance group show that the direct and indirect effects of health shock on the poverty vulnerability of rural chronic disease families are significant. Their action directions are opposite, and the final total effect is positive reinforcement. In addition, family coping ability and community health support system have a significant direct impact on the poverty vulnerability of rural chronic disease families, and both have a positive impact. For the poverty avoidance group, health impact can affect poverty vulnerability in two ways: one is the direct effect caused by various acute and chronic diseases, disability, mental disorders, and other diseases. Second, through the intermediary role of a family coping ability dimension and community health support system dimension, the health risk is transformed into economic risk and finally manifested as poverty vulnerability.

In terms of the absolute value of the total effect, the dimension of family coping ability has the greatest effect on the poverty vulnerability of rural chronic disease families. That is, the stronger the family coping ability, the more inclined it is to avoid poverty vulnerability. The influence of health impact and community support system is small. This result indicates that family coping ability is the main reason why the poverty avoidance group can avoid the vulnerability of current and expected poverty.

The results of the structural equation model analysis of potential poverty group show that the direct and indirect effects of health shock on the poverty vulnerability of rural chronic disease families are significant. The action direction of the two is opposite, and the final total effect is negative weakening. The dimension of family coping ability also has a significant direct effect on the poverty vulnerability of rural elderly families with chronic diseases, and its direction is negative. However, the impact of the community health support system on the poverty vulnerability of rural elderly families with chronic diseases is not statistically significant. Therefore, for the potential poverty group, the path of health impact on poverty vulnerability mainly includes the following: first, the direct effect caused by various acute and chronic diseases, disability, mental disorders and other diseases; second, through the intermediary role of family coping ability dimension, the health risk is transformed into economic risk and finally manifested as poverty vulnerability.

In terms of the absolute value of the total effect, the dimension of family coping ability has the greatest effect on the poverty vulnerability of rural chronic disease families. That is, the weaker the family coping ability, the more likely it is to have poverty vulnerability. The second is the dimension of health impact. As the direction of its direct and indirect effects is inconsistent, the absolute value of the final total effect is small.

The results of the structural equation model of the temporary poverty group show that the direct effect of health shock on poverty vulnerability is not statistically significant but is significant through the intermediary effect of family coping ability. For the temporary poverty group, health risk factors do not directly lead to poverty vulnerability but lead to poverty vulnerability through the intermediary role of family coping ability.

The structural equation analysis results of the persistent poverty group show that the direct and indirect effects of health shock on the poverty vulnerability of rural chronic disease families are not significant. However, the total effect is significant, and the total effect shows a positive strengthening effect. Family coping ability and community health support system have a significant direct impact on the poverty vulnerability of rural chronic disease families, and both have a positive impact. For the persistent poverty group, the path of health impact on poverty vulnerability is not the direct negative effect caused by health problems. However, the intermediary role of family response and community support transform health impact into economic risk and finally fall into poverty vulnerability.

## Discussion

In this study, an inconsistency was found between poverty vulnerability and actual poverty, which was reflected by the high incidence of poverty among people with low vulnerabilities. The structural equation modeling of different vulnerability types showed that different groups of health risk factors have different paths. As a predisposing factor, health shock had a significant direct impact except for the persistent and the temporary poverty groups. The family coping ability played an important role in each classification. The community health support system was not significant in the potential poverty group and the temporary poverty group. The possible reasons for the above results are as follows. The preferential medical policies for the low-income households and the coping attitude to health risk may be potential factors for the inconsistency between poverty vulnerability and actual poverty. The multidimensional nature of poverty and the negative impact of health shock can be solved by family response measures in time. This result, may explain why the persistent and temporary poverty groups are not directly affected by the negative effects of health shock itself. Family coping capacity and external security policy are the main and auxiliary forces to avoid current poverty and poverty vulnerability, respectively.

The preferential medical policies for high vulnerability households may be a potential factor causing the inconsistency between poverty vulnerability and actual poverty. People at a high risk of poverty may receive interventions to prevent them from falling into poverty. Those at a low risk of poverty may lack interventions to drive them into poverty. In China, many medical policies are provided for low-income households. In the era of poverty alleviation, many policy tilts are available for the poor on the basis of basic medical insurance. For example, China uses a social health insurance program and a medical financial assistance program to protect the poor from the financial risks of illness (42, 43). The two key objectives of New Cooperative Medical Schemes are to promote the utilization of health services among the rural population and provide financial protection against catastrophic expenditures for rural households. In addition, as one of the government schemes to achieve universal health coverage, New Cooperative Medical Schemes aims to benefit the whole rural population including the poor and vulnerable, and improving healthcare equity (44). The medical financial assistance program in China, targeting low-income households, is used as a supplement to social health insurance programs. This program provides extra financial assistance to low-income households in addition to social health insurance to protect them from catastrophic health expenditure (42).

The coping attitude to health risk may be the reason for the high incidence of real poverty in families with low vulnerability. Families with high vulnerability to poverty may pay attention and take active measures to avoid health risks, such as purchasing medical insurance. By contrast, families with low vulnerability do not pay enough attention to health risks and do not take measures to prevent health risks. Therefore, once suffering from health shock, those families are likely to fall into poverty.

The multidimensional nature of poverty may be the reason why the persistent poverty group is not directly affected by the negative effects of health shock itself. Poverty is multidimensional, and health shock is one of the factors leading to poverty vulnerability. Persistent poverty is mainly because of the lack of basic material needs and low long-term income. In addition, the extremely weak working ability, learning ability, social support, and ability to deal with external risks all result in the lack of sustainable income-generating ability (45). In the long run, the marginal vulnerable groups have formed in the society, and the passage of time of poverty is likely to cause extreme poverty (46). In addition, most of these families live in poor rural areas with inconvenient transportation. The construction of a community health support system is seriously lagging behind, and the accessibility and availability of medical services are insufficient (47, 48). The above factors place these families in a state of long-term income deficit. Even if they do not suffer from health shock, they are also limited to poverty and poverty vulnerability because of the lack of coping ability and imperfect community support system.

The negative impact of health shock can be solved by family response measures in time, which may be the reason why the temporary poverty group is not directly affected by the negative effects of health shock itself. The temporary poverty group is less suffering from very serious and lasting health shock, and more from mild and acute disease shock. Most of them fall into the state of poverty for a short time because of the fast course and short time. However, the decline of health status can be quickly alleviated by a strong family coping ability (49). Therefore, for these two types of families, health shock is more the inducing factor of poverty vulnerability than the direct influencing factor. However, for other types of families, health shock is not only the inducing factor of poverty vulnerability, but also the direct influencing factor.

Family coping ability is the main force to avoid current poverty and poverty vulnerabilities. The external guarantee policy is an auxiliary force to avoid the current poverty and poverty vulnerabilities. In the face of health shock, families with different income levels adopt different coping strategies, which is also an important reason for different poverty vulnerability outcomes. Specifically, high-income families often use relatives and friends to help, use cash/deposits, loans, and increase income sources to tide over family economic risks. By contrast, low and middle-income families are more likely to deal with them by selling their properties and reducing living expenses (50). Evidently, these strategies bring great pressure on the economic life of low-income families, and they are difficult to alleviate effectively in a short time. The insufficient accumulation of family wealth largely limits the possibility and feasibility of families to take appropriate measures, thereby preventing them from successfully avoiding the vulnerability of current and expected poverty. Pension and medical insurance can play a certain role in alleviating the health poverty of the rural elderly (51). Therefore, an institutionalized external security policy is important to alleviate the vulnerability of poverty caused by health shock.

The present study had some limitations. First, the retrospective data used in this study may be disturbed by the recall bias, which makes the analysis results biased with the actual situation. Second, the definition of the family population in this study is based on the permanent resident population (family members who live at home for more than 6 months each year, and students studying in other places) as the standard. However, in the actual situation in my country's rural areas, many children work in other places all year round. The grandchildren support the elderly, which may cause the residential and economic relations of these families to be inconsistent. Third, the result of poverty vulnerability in this study is much higher than that of the 8% poverty vulnerability rate of chronic disease patients measured by relevant scholars with the standard of $2 per day poverty line [52]. The main reasons are as follows. First, the poverty line standard selected in this study is lower than that in this study. Second, the data of this study only included rural families in poverty-stricken areas of central and western China, and the data of the above study covered the rural and urban samples of the whole country. Therefore, the health capital stock and the ability to resist the economic risk of disease of the sample are generally strong, making the poverty vulnerability level measured in this study high.

## Conclusion

Even if absolute poverty is eliminated, the families of rural elderly with chronic diseases are extremely easy to fall into poverty or poverty vulnerability again because of their limited income generating capacity, low social support, and fragile health status. The social and economic environment in rural areas should be improved in a diversified manner, the income of farmers should be increased, and the social support and the health status of the elderly should be improved. Refined management of health risk factors should be conducted, and differentiated intervention measures should be adopted for potentially poor households and persistently poor households.

Family coping ability plays an important role in avoiding current poverty and the vulnerability of expected poverty. Family response measures are the main force to avoid poverty vulnerability, and external security policies are the auxiliary force to avoid poverty vulnerability. Therefore, the income-generating ability of rural households based on the actual local conditions must be improved. First, the initiative of farmers must be simulated, and they must be guided to establish their own sense of poverty relief. Second, relevant departments should formulate policies to benefit farmers and provide them preferential policies for sustainable development. Finally, common diseases in various regions should be focused on through health management, promoting healthy lifestyles and other related measures, targeted interventions on health risk factors, and health diagnosis and treatment management.

The health risk factors and their action paths of different vulnerability types have similarities and differences. Health shock is a direct factor to avoid poverty, and the mediating effect of family and community is significantly different among different vulnerability types. Therefore, the prevention and control of poverty vulnerabilities should be diversified and targeted. Policies implemented should be based on people and localities, the causes of poverty and returning to poverty, and the types of poverty vulnerabilities. Classified management and multi-path collaborative governance should be adopted.

The use efficiency of medical insurance should be improved further, and the responsibility of medical insurance targeted poverty alleviation must be clarified. The use and management of the basic medical insurance fund follow the principle of “fixed expenditure based on income, balance of revenue and expenditure and slight balance.” This principle requires appropriate control of the fund and cumulative balance rates in the current year and strives to achieve an effective balance between fund risk control and increasing security. Moreover, this principle ensures that the overall financing and security levels of basic medical insurance adapt to the development of economy and society. We also need to improve the turnover efficiency of a medical insurance fund. Meanwhile, the policy of paying after diagnosis and treatment and the “one-stop” real-time settlement model for rural poor patients in the county have been implemented. The objectives are to eliminate the obstacles of poor people to medical treatment and ensure that rural poor patients are treated in time, “affordable” and “convenient.” Therefore, we should improve the allocation efficiency of medical insurance funds, shorten the allocation cycle, and explore clear provisions on the allocation proportion of relevant funds of designated medical institutions in a certain year. From another aspect, for the poor patients who cannot settle the out-of-pocket expenses at one time when they are discharged, the installment payment mode can be adopted. The poor patients who maliciously default on the expenses can be included in the blacklist and disqualified from enjoying the health poverty alleviation policy. The inclusive function of basic medical insurance, the inclined anti-poverty function of serious illness medical insurance, the bottom-line guarantee function of medical assistance, and the supplementary function of commercial insurance can be maximized by continuously strengthening the supporting policies and measures related to health poverty alleviation. This notion reflects that medical insurance, as a key force of health targeted poverty alleviation, plays a role in transferring low-income people to truly see and afford to see a doctor.

## Data Availability Statement

The raw data supporting the conclusions of this article will be made available by the authors, without undue reservation.

## Ethics Statement

Written informed consent was obtained from the individual(s) for the publication of any potentially identifiable images or data included in this article.

## Author Contributions

YM and QX initiated the study, managed the data collection, performed the data analysis, and wrote the first draft of the manuscript. CY, HL, and JW contributed to its design, reviewed critically subsequent drafts of the manuscript, and approved its final version. All authors read and approved the final manuscript.

## Funding

This study was funded by (1) The National Natural Science Foundation of China (72074086); (2) The National Natural Science Foundation of China (71673093); (3) Humanities and Social Sciences of Ministry of Education Planning Fund (16YJA840013).

## Conflict of Interest

The authors declare that the research was conducted in the absence of any commercial or financial relationships that could be construed as a potential conflict of interest.

## Publisher's Note

All claims expressed in this article are solely those of the authors and do not necessarily represent those of their affiliated organizations, or those of the publisher, the editors and the reviewers. Any product that may be evaluated in this article, or claim that may be made by its manufacturer, is not guaranteed or endorsed by the publisher.
